# Biologically Informed Individual-Based Network Model for Rift Valley Fever in the US and Evaluation of Mitigation Strategies

**DOI:** 10.1371/journal.pone.0162759

**Published:** 2016-09-23

**Authors:** Caterina M. Scoglio, Claudio Bosca, Mahbubul H. Riad, Faryad D. Sahneh, Seth C. Britch, Lee W. Cohnstaedt, Kenneth J. Linthicum

**Affiliations:** 1 Department of Electrical and Computer Engineering, Kansas State University, Manhattan, KS, United States of America; 2 USDA-Agricultural Research Service Center for Medical, Agricultural, and Veterinary Entomology, Gainesville, FL, United States of America; 3 USDA-Agricultural Research Service Center for Grain and Animal Health Research, Manhattan, KS, United States of America; 4 Department of Electronic Engineering, "La Sapienza" University of Rome, Rome, Italy; Division of Clinical Research, UNITED STATES

## Abstract

Rift Valley fever (RVF) is a zoonotic disease endemic in sub-Saharan Africa with periodic outbreaks in human and animal populations. Mosquitoes are the primary disease vectors; however, Rift Valley fever virus (RVFV) can also spread by direct contact with infected tissues. The transmission cycle is complex, involving humans, livestock, and multiple species of mosquitoes. The epidemiology of RVFV in endemic areas is strongly affected by climatic conditions and environmental variables. In this research, we adapt and use a network-based modeling framework to simulate the transmission of RVFV among hypothetical cattle operations in Kansas, US. Our model considers geo-located livestock populations at the individual level while incorporating the role of mosquito populations and the environment at a coarse resolution. Extensive simulations show the flexibility of our modeling framework when applied to specific scenarios to quantitatively evaluate the efficacy of mosquito control and livestock movement regulations in reducing the extent and intensity of RVF outbreaks in the United States.

## Introduction

Rift Valley fever (RVF) is a viral zoonosis endemic to sub-Saharan Africa, Egypt, and the Arabian Peninsula that primarily affects livestock–mainly sheep, goats, cattle, and camels–but also has the capacity to infect humans [1]. Infection can cause severe disease in both livestock and humans. The disease can also result in significant economic losses due to death and abortion among Rift Valley fever virus (RVFV)-infected livestock, and trade restrictions in affected countries. Mosquitoes are the primary disease vectors; however, RVFV can also spread by direct contact with infected tissues [2]. In the endemic range of RVF, once livestock are infected by primary transovarially-infected *Aedes* species mosquitoes, a variety of secondary *Culex* and *Mansonia* species mosquitoes may transmit RVFV to humans and other mammals, spreading the disease [3, 4]. *Aedes*, *Culex*, and *Mansonia* genera of mosquitoes are thought to be the main RVFV vectors due to virus isolation in nature and their high vector competence. Livestock movements, typically motivated by trading and marketing purposes, can accelerate the transmission of RVFV between herds of animals that are separated by large distances [1]. An epizootic of RVF is generally observed in Africa during years in which heavy rainfall and localized flooding occur [5]. In fact, rainfall causes ground pool *Aedes* mosquito eggs in the soil, many of which are already infected with RVFV, to hatch. Non-transovarially infected *Aedes* mosquitoes may acquire the virus from feeding on infected animals, and may potentially transmit the virus vertically, so that new generations of infected mosquitoes may hatch from their eggs. Vertical transmission of the virus between generations of *Aedes* mosquitoes, and the capability of virus-infected *Aedes* eggs to survive for up to several years through dry conditions, provides a potential mechanism for maintaining the virus through inter-epizootic periods [6].

The first cases of RVF were identified in Kenya in 1931 [7] during an investigation into abortion among sheep on a farm in the Rift Valley near Lake Naivasha. Although the incidence of human infection with RVFV was reported soon after the identification of the virus in 1931 [7], the first major outbreak in humans was not reported until 1951. In that year, approximately 20,000 people were infected during an outbreak of RVF in cattle and sheep in South Africa [8]. The RVF virus is generally found through regions of Eastern, Western, and Southern Africa, and less frequently in the Nile Delta region of Egypt, where sheep and cattle are present. The first RVF outbreak outside Africa was reported in Saudi Arabia and Yemen in 2000–2001 [9]. There are great concerns that RVFV could also spread to Europe and the Western hemisphere. In the United States (US), the risk of introduction and establishment of RVFV is not negligible, and increasing international travel and future climate changes can further elevate this risk [10]. Computer modeling of RVFV propagation can help in understanding the characteristics of a potential spreading process in the US and in developing effective mitigation strategies. Over the last decade, many models have been developed to study and analyze the possible transmission and spread of RVFV in the US as well as in endemic regions of Africa. One of the first models was developed by Favier et al. [11] to model the role of space in RVF endemicity in West Africa. In this model, a pond is placed under surveillance with a one year step and with an associated population of domestic animals and infected mosquito eggs. The population of domestic animals is divided into *N* classes of age: the first class represents the juvenile one while the others are adult. The pond consists of *L* layers where infected eggs can lie. Two immunological states are considered—Susceptible and Resistant. If the number of emerging infected vectors is greater than a threshold value and if there are susceptible adult animals, an epidemic is triggered. At the time of an epidemic, more infected eggs are produced. This model aims to calculate the total number of infected eggs, the total number of abortions of sheep, and the total number of infected livestock age classes.

A model proposed by Gaff et al. [12] considers two mosquito populations, *Aedes* and *Culex—*modeled using the Susceptible-Exposed-Infected (SEI) model—and a population of livestock animals—modeled using the Susceptible-Exposed-Infected-Recovery (SEIR) model. The structure of the model of the two mosquito species is similar, but it differs in one aspect: *Aedes* species has two compartments in addition to SEI, namely, Infected Eggs and Uninfected Eggs. Infected eggs are not included in the *Culex* species epidemic model, since these mosquitoes most likely cannot transmit RVFV vertically. The model proposed by Mpeshe et al. [13] is an elaboration of Gaff et al. [12] with an additional human host, however, considering only one species of mosquitoes. Therefore, they consider three populations in their model: mosquitoes, livestock, and humans. Similar to the model of Gaff et al., the models for mosquito populations and for livestock populations in Mpeshe et al. are SEI and SEIR, respectively. In this model, animals can infect humans. The human model is similar to the animal model, but humans cannot infect mosquitoes or livestock. The models of Xue et al. [13, 14] are a combination of Mpeshe et al. [13] and Gaff et al. [12] and consider both *Aedes* and *Culex* mosquitoes, livestock, and humans. Not only are the models of Xue et al. more comprehensive because they include all four populations involved in RVF epidemiology in endemic areas, but they are also unique for they include spatial movements of these four populations. This model is based on weighted movement networks: network nodes represent geographical regions, and the weights represent the level of contact between regional pairings for each set of species. In [14], this model is applied to an RVF outbreak in South Africa, while in [15] it is applied to a hypothetical scenario in the US. The model can differentiate the maximum number of infected individuals among different locations and can simulate the spatio-temporal evolution of the outbreak in multiple locations.

Chitnis et al. [16] consider in their model only one type of mosquito (one species in the Genus *Aedes*). This class is divided into three compartments: Susceptible, Exposed, and Infected. In addition to this class, this model considers the cattle population divided into four compartments. Unlike other models, cattle are represented by the Susceptible-Asymptomatic-Infectious-Recovered model, which means some cattle (which do not exhibit clinical signals apart from abortion) transmit the virus at a lower rate than other cattle, which have acute clinical signs. To represent vertical transmission in their extended model, they added two new compartments in the mosquito population. These new compartments represent susceptible and infected juvenile mosquitoes. This model uses environmental parameters from both East and West Africa.

Another model proposed by Gao et al. [17] aims at modeling the movement of animals. This is a three-patch model: Sudan (patch 1)—Nile (Patch 2)—feast (Patch 3), and animal movement is constrained from patch 1 to patch 2, and then from patch 2 to patch 3. The aim of this paper was to provide evidence that a major epidemic can occur when an importation of infected livestock is coincident with high mosquito density.

Barker et al. [18] developed a model consisting of three populations (*Aedes* mosquitoes, *Culex* mosquitoes, and livestock hosts). Mosquitoes are represented with an SEI model while livestock populations are represented with an SEIR model. In this model, similar to that of Gaff et al. [12], vertical transmission is considered for *Aedes* mosquitoes. This model is applied to an agricultural region of California, US, to verify its validity.

One of the latest models is proposed by Chamchod et al. [19]. Authors aim to investigate the emergence of an RVFV outbreak and epizootic and enzootic cycles in disease free regions. In their model, the human population is not considered, and cattle are divided into three compartments: Susceptible, Infectious, and Recovered. Mosquitoes are considered to be uninfected or infectious.

Together, these models offer multiple viewpoints on the very complex system of RVFV epidemiology in endemic and emerging regions. However, a major drawback of these models in a simulative scenario analysis is that they require the estimation of a large number of unknown parameters. Accurately estimating such parameters is challenging and prone to large error in practice.

In this paper we develop a model for a scenario of RVFV epidemiology in the US where only one population–the cattle population–is represented at the individual animal level through a movement network, while mosquito impact is given in an aggregated way by the value of one parameter proportional to the vectorial capacity of focal putative US mosquito vectors of RVFV. Vectorial capacity is a collective measurement of the efficiency of vector-borne disease transmission. It takes into account mosquito vector density with respect to hosts, daily probability of a host being fed upon, probability of daily survival of the vector, length of the virus extrinsic incubation period, and vector competence. Overall, we analyze the impact of two modeling aspects on the epidemic evolution: (1) the cattle movement network, implicitly representing the probability of cows moving from their origin farm to a new destination farm, and (2) the mosquito vectorial capacity, which represents mosquito abundance and vector competence for RVFV. One of the advantages of our model is that it begins with the simple scenario that an RVFV-infected cow appears on a farm, and does not attempt to impose the epidemiology of the virus from endemic regions. This scenario is plausible because the initial conditions leading to RVFV appearing in the US could be a highly localized series of transmission events involving, for instance, a viremic human recently returned to the US from an RVF-endemic country. A very limited number of US mosquitoes could feed on that person, become infected with RVFV, and after an appropriate time amplifying the virus then transmit the virus while feeding on cattle, deer, elk, or sheep, for example.

Thus in our model, we do not introduce unknown (and unknowable) parameters of myriad possibilities common in endemic regions of sequential *Aedes*/*Culex* infections or transovarial mosquito harborage of RVFV. We simply look at the initial farm (the index case location), make key guided assumptions of the potential for local mosquitoes to become infectious from the initial, single cow and then look at how this farm could produce a set of infected cows that become infectious *en route* to other farms and in turn infect mosquitoes on other farms which then infect more cows. One important assumption is that cows are moved before they look sick or are infectious and that a series of movements could plausibly take place among farms/operations before people realized an RVF outbreak was in its early stages and conducted a mass stop-movement. Another key to simplicity is that the scenario could take place within a single generation of mosquitoes, or closely overlapping cohorts of adults that provide an ongoing opportunity for infectious mosquitoes, obviating the need to include vertical transmission from mosquito to mosquito or inter-seasonal virus maintenance in the model.

We model RVFV in the state of Kansas because there are potentially competent RVFV vectors and potentially susceptible cattle both present in high numbers in Kansas with abundant, realistic expectations of connectivity across largely homogeneous habitat, and connectivity to other cattle areas of the US—important for future modeling of the larger picture of RVFV transmission risk outside Kansas, with a Kansas origin. Furthermore, there is a general lack of mosquito control operations Kansas which increases the risk of transmission of RVFV should it be introduced, compared to other cattle-producing states that may have more structured mosquito surveillance and control infrastructure. From the modeling perspective, Kansas is suitable also because of the absence of mosquitoes in most winter months which provides natural starting and stopping points for modeling mosquito-borne RVFV transmission; in contrast to Florida, for instance, with year-round mosquito populations. Although eastern US coastal areas do have connectivity with RVFV-endemic areas via sea and airports [20], the final destination of for example human travelers harboring RVFV or containers with RVFV-infected mosquitoes could very well be far inland and be reached very rapidly from these ports [20, 21]. Future similar modeling studies may be conducted for other ecological regions of the US encompassing states with high production of cattle and other RVF-susceptible livestock that also have long-term mosquito population surveillance records. Results from these future studies will be compared to results from the Kansas model to provide a basis for calibration of the framework by ecological region.

We first develop the simulation model using the generalized epidemic modeling framework (GEMF) developed by the Network Science and Engineering (NetSE) group at Kansas State University [22] and successively describe the data we have used in this simulation study. In particular, we have used farm sizes and locations from Bai et al. [23], movement data from Schumm et al. [24], and mosquito data from Turell et al. [25–30]. We then consider other movement networks generated by mathematical models, namely exponential and power law, and two different farm organizations: fully connected and partitioned. Under these different scenarios, we carry out extensive simulations to test the effectiveness of mosquito control and movement regulations and restrictions. In summary, our aim is to show the quantitative efficacy of interventions strongly depends on specific movement levels and mosquito vectorial capacity, ranging from a negligible value to a significant reduction of the total final infected population.

## Materials and Methods

In the following, we introduce the network-based individual-level model developed for the simulations and the used data.

### Model

**Fig 1:**
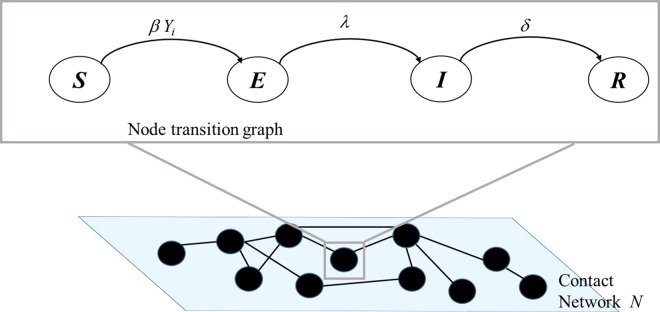
Network-based individual SEIR model.

In this paper, we adapt and use GEMF developed by Sahneh et al. [22], a very flexible tool capable of simulations of infectious disease models with multiple numbers of compartments and multiple network layers of interaction. To model RVFV transmission, we consider a spreading process of a pathogen among *N* nodes, where *N* is the total number of individuals in the cattle population. Each node can be in one of four compartments *Susceptible*, *Exposed*, *Infected*, and *Recovered*. We represent individual cows as nodes in a network; the number of nodes in this network is equal to the cattle population size, *N*. Links among nodes represent the possibility of RVFV transmission from an infected cow to a susceptible cow by a mosquito. Fig 1 shows an example of our SEIR model on a movement network.

The effective mosquito population size in this model is governed by the vectorial capacity of the particular mosquito species present; i.e., for various reasons not all individual mosquitoes of a population of a potentially competent vector species for RVFV will transmit RVFV to cattle on the farm. Vectorial capacity is a composite index of a mosquito species that takes into account not only vector competence but also daily survival and population density, and can be estimated using real-world data. *Vector competence* itself is an index that captures the fact that not all individuals of a competent mosquito species will necessarily become infected by the virus when exposed to it by blood feeding on an infected cow, and of those that do become infected by the virus, not all of them will go on to transmit the virus (i.e., develop a salivary gland infection and be infectious) to naïve cows during subsequent blood-feeding activity. If non-infected competent mosquito vectors are present and feed on a newly-arrived infected cow, some of these mosquitoes will become infected with RVFV and after a period of viral replication and migration to the salivary glands within these mosquitoes, some will be able to transmit the virus to naïve cattle by bite during blood-feeding, thus spreading the infection. Otherwise, the virus remains isolated within the initially infected cow that arrived on the farm and cannot spread to other individual cows on that farm. The more infected cows that arrive at an uninfected farm, the more likely that the local competent mosquito population will contact infectious cows and develop a proportion of infectious mosquitoes (governed by the vectorial capacity of that mosquito species) that can more quickly spread the virus to naïve cows at that farm. The model takes into account the time delay for cows to become viremic after exposure to the infectious mosquito. This delay is considered during the movement phases of the model where cows move between/among farms.

In this model, the infection can spread if a susceptible node is in contact with at least one infected node. In particular, an infected cow (node 1) can transmit RVFV to a susceptible cow (node 2) only if there are enough RVFV-competent mosquitoes to first bite the infected cow (node 1) and, after an appropriate period of viral replication in the mosquito, successively bite the susceptible cow (node 2). A link between node 1 and node 2 represents the possibility of virus transfer via a mosquito. The susceptible node 2 can become exposed due to its link with node 1 with a transition rate *β*. We assume that the rate *β* is proportional to the vectorial capacity. Since infection processes are statistically independent, the transition rate for a susceptible node to the exposed state is the infection rate *β* times the number of infected neighbor nodes *Y*_*i*_. In other words, the total rate at which a cow can become infected is proportional to the infected cows in the neighborhood and the population size (or density) of competent mosquito vectors. The exposed compartment represents the delays for a susceptible cow to become infectious. An exposed node will then become infectious with a rate λ and, finally, it will transition into the recovered/removed and immune state. This last transition happens with recovery rate *δ*.

In the SEIR model based on GEMF, the time distributions of processes determining the infection events of a node are assumed to be exponentially distributed and statistically independent from each other. The node-level Markov process for node *i*, *i* = 1, 2, … *N*, is expressed as:

$$\mathrm{Pr}[x_{i}(t+\Delta t)=1|x_{i}(t)=0,X(t)]=\beta Y_{i}\Delta t+o(\Delta t),$$

$$\mathrm{Pr}[x_{i}(t+\Delta t)=2|x_{i}(t)=1,X(t)]=\lambda \Delta t+o(\Delta t),$$

$$\mathrm{Pr}[x_{i}(t+\Delta t)=3|x_{i}(t)=2,X(t)]=\delta \Delta t+o(\Delta t),$$

where *x*_*i*_ = 0, 1, 2, 3 corresponds to node *i* being in the susceptible, exposed, infectious, recovered/removed state, respectively. The value *X*(*t*) is the joint state of all nodes–the network state–at time *t*, and *Yi* is the number of infected neighbors of node *i*. GEMF simulates iterations of the stochastic Markov process corresponding to the epidemic model. Each simulation is event-based and stops either when the number of events or the simulation time reaches a maximum value. Individual-based models are simulations based on the global consequences of processes involving individuals of a population. In these models, the characteristics of each individual are tracked through time. Individual-level models provide more accurate predictions than meta-population models [31]. However, often the data to inform individual-level models are not available. Our model has the flexibility to be equivalent to a meta-population model, by representing the cattle network within a farm as a fully connected network. However, it also has the potential to incorporate more complex topologies for contacts within the farm, when individual animal contact data are available.

#### Network structure

**Fig 2:**
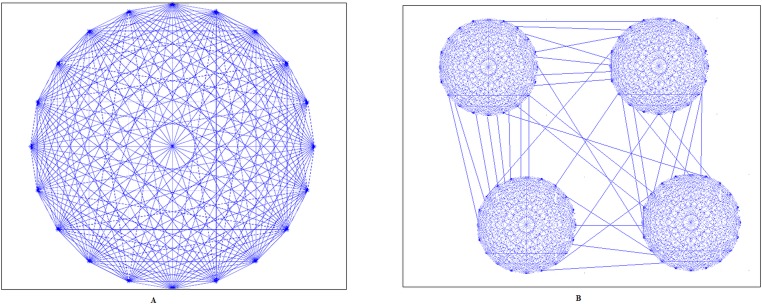
Cattle contact network inside a farm with intra-farm links. (A) The farm is fully connected. (B) The farm is partitioned.

A realistic representation of RVF requires appropriate network structure. One possible assumption is that cattle from the same farm can all be in contact, so a fully connected network can represent the contacts among animals within the same farm. We call these links inside a farm intra-farm links. This case is shown in Fig 2A. Conversely, Fig 2B represents the contact network when the farm is partitioned. It should be noted here that the contact between cattle, where RVFV transmission is concerned, is via a mosquito picking up the virus from one cow and then infecting another cow by bite during blood feeding. There is not direct transmission of RVFV from one cow to another in this contact network.

**Fig 3:**
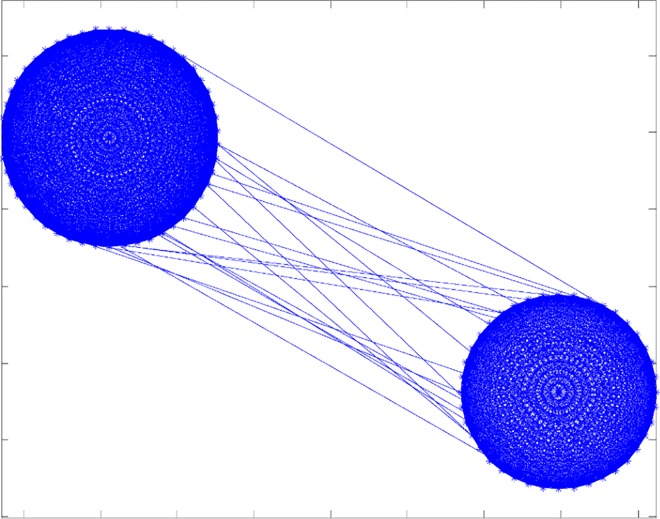
Inter-farm links between two farms.

When simulating a geographical region with several farms with specific sizes, each farm can be represented as a network presented in Fig 2. Regarding links between farms (inter-farm links), they can implicitly represent cattle contacts due to movements; movement can be of infected or uninfected cows, but for this model (this scenario) we assume that mosquito populations, which include infectious mosquitoes, are confined to each farm and do not disperse between or among farms. Assuming abundant hosts in any given farm, few infected mosquitoes will likely be compelled to disperse to seek hosts elsewhere [32, 33]. Also, wind-mediated dispersal or accidental human-mediated movement of infected mosquitoes for instance via aircraft or road vehicles—which are responsible for rare events of introduction in a new geographical location—are not expected to be important contributions to the epidemiological network when considering the propagation after the initial introduction. An example of a network with two farms and several inter-farm links is shown in Fig 3.

Inter-farm links can be obtained by movement data or by an estimation of the movement probabilities when direct data are missing. In this paper, we use the estimated movement probabilities of Schumm et al. [24] obtained by formulating and solving a large, convex optimization problem. This approach used as input the optimization cattle population and aggregated movement data from the United States Department of Agriculture’s database and optimally estimated non-disclosed data points to construct a complete database of inputs. The movement probability estimation is then formulated as a convex optimization problem maximizing an entropy objective function, subject to a flexible set of linear constraints with minimal assumptions. The solution results produce county-to-county movement probabilities among stratified subpopulations at the farm level, as well as birth and slaughter rates of cattle for 1034 counties of 10 central states of the United States.

Following the structure of the cattle industry operation in the US, farms are divided into three classes: those that primarily produce calves, those that then fatten/grow these calves into beef cows (preslaughter), and dairy cows. Additional constraints coming from the cattle industry structure considered by Schumm et al. as constraints of the optimization problem are:

- There are no outgoing movements from preslaughter feed programs except for the outgoing movements of cattle for slaughter.
- Cattle classified as dairy cattle do not move into preslaughter feed programs.
- Populations of preslaughter feed cattle having population sizes of 200 head of cattle or more are responsible for all shipments to slaughter that result in yearly totals of 500 or more head shipped from a single premise.
- All sub-populations remain constant on a year-to-year basis.

**Fig 4:**
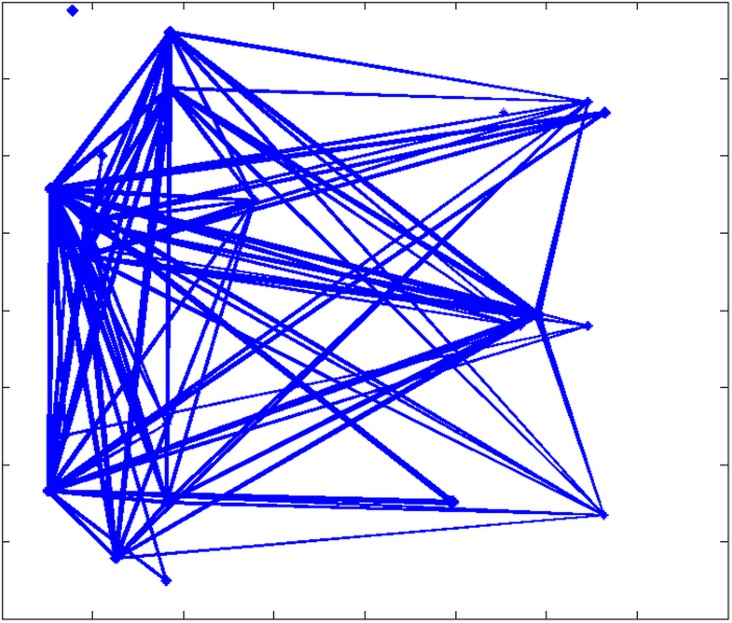
Inter-farm links from estimated movement among 27 farms in Riley County, Kansas.

Fig 4 illustrates one movement network created using these estimated movement probabilities. Links of the network can represent direct movements or via sale barns. Using these estimated data, cows are not randomly moved from one farm to another but follow the industry operation basic structure.

Calf operations, beef operations, dairy operations, and sale barns are locations where cattle are concentrated in space and time which produce opportunities for mosquitoes to become infected by an infectious cow and then spread the virus to nearby naïve cows. The size (in number of cows) of the index case farm and the vectorial capacity of mosquitoes at that farm will govern the extent and speed at which the single arriving infectious cow will, via mosquitoes, lead to RVFV infections appearing in susceptible cows at that farm, which will, in turn, govern the proportion of infectious cows moving from that farm to another farm, and so on. Movement of infectious cows assumes that RVFV infection was not detected, which is most likely immediately after being bitten by infected mosquitoes and before viral amplification in the cows that would accompany (cause) visible symptoms.

**Fig 5:**
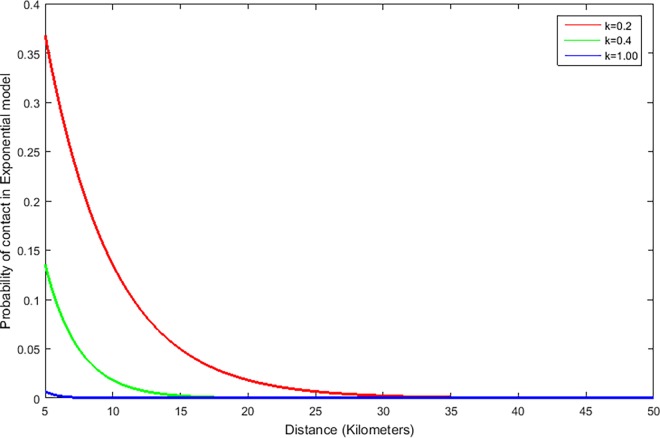
The probability of link creation in an exponential model with increasing distance for *k* = 0.2, 0.4, and 1.0 km^-1^.

When movement data are missing, one way to create inter-farm links is to consider a mathematical function to generate links among farms at a given distance *d*. In other words, the probability *p* of generating links can be driven by an *exponential function* and set equal to *p* = *e*^-*kd*^, where *k* is a parameter and *d* is the distance between two farms. In this way, the infection spreads in the neighboring farms, with the ability to expand also in a larger area. Fig 5 represents how the probability of creating links between animals in different farms in an exponential model changes with distance *d* and the parameter *k*. Distance here is measured in kilometers (km) and the parameter *k* in km^-1^. We chose the values of *k* in such a way that they represent three real life scenarios of having a high (*k* = 0.2 km^-1^), medium (*k* = 0.4 km^-1^), or low (*k* = 1 km^-1^) probability of contact.

Another way to generate inter-farm links is to consider a probability *p* = (*d*/*D*_*min*_)^*-h*^, where *d* is the distance between two farms, *D*_*min*_ is the parameter, and *h* is the power coefficient. This second model follows a power law function, which is characterized by a greater chance of creating links over long distances, compared to more limited chances of creating links over the same long distances in the exponential model.

**Fig 6:**
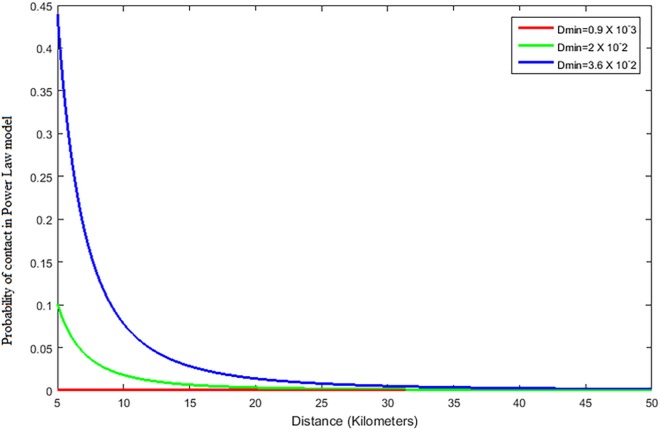
The probability of link creation in power law model with increasing distance for *D*_*min*_ *=* 0.9 x 10^−3^, 2 x 10^−2^ and 3.6 x 10^−2^ km.

In Fig 6, which shows the probabilities of creating links among farms with a power law model, we can see that probabilities follow a trend similar to the one of the exponential model. The probability of link creation is much higher in this model for the same given distance so that links can be created between more distant animals than in the exponential model. While choosing the value of *D*_*min*_ and *h*, we tried to select some values that create a similar number of links between animals as in the exponential model-based networks. These values are *D*_*min*_, = 0.9 x 10^−3^ km, corresponding to *k* = 1 km^-1^, *D*_*min*_ = 2 x 10^−2^ km, corresponding to *k* = 0.4 km^-1^, and *D*_*min*_, = 3.6 x 10^−2^ km, corresponding to *k* = 0.2 km^-1^. The value of *h* was chosen as 2.5 invariably for all simulations.

#### Parameters

In our SEIR network model, there are four parameters to be set: *λ*, *δ*, and *β*; and one parameter for the contact network model: *k* for the exponential model, or *h* for the power law. We can consider reasonable values for some of these parameters. In particular, we know that signs and symptoms of RVF in cattle (a period assumed to be infectious to a mosquito) persist for about one week, following a 2–3 day incubation period following a bite from an infectious mosquito and successful transfer of virus to the cow [34]. In other words, two realistic values of these parameters are *δ* = 0.14 day^-1^ and *λ* = 0.33 day^-1^. For *β*, we use the vectorial capacity as proposed by Garret-Jones [35], and explained below in the section *Mosquito abundance and* vectorial capacity. For the purpose of performing a sensitivity analysis, we also use in our simulations the values for *β* set by several existing models [9, 34] which assume a range from 0.0021 to 0.2762. The last values to be set are *k* and *h*. Recalling that *k* is the parameter that allows us to calculate the probability of generating links among farms, three interesting values considered in this study are *k* = 0.2, 0.4, or 1 km^-1^. For a power law network model, we select an exponent *h* = 2.5, and will select *D*_*min*_ such that the total number of links generated will be comparable with a corresponding exponentially generated networks.

### Data

#### Farm location and sizes

Accurate information on farm locations and sizes are necessary for a realistic model. Unfortunately, little information is publicly available due to privacy constraints for many US farms. However, a report from the Kansas Department of Transportation [23] on transportation logistics and economics of the processed meat and related industries in southwest Kansas has in Appendix IV a detailed description of feed yards and cow-calf farms in Kansas. This database contains coordinates of 1667 farms and their sizes. For our simulations, we selected 27 farms with 7687 cattle total around Riley County, Kansas.

#### Cattle movement network from data

A cattle movement network among these 27 selected farms can be determined using the cattle movement parameters estimated by Schumm et al. in [24]. In this publication, Schumm and colleagues used aggregated data from the US Department of Agriculture to estimate the detailed movement of cattle among nine classes of farms on the basis of their size regarding the number of cattle (small, medium, or large) and their type (dairy, beef, or preslaughter). The estimation was performed for the ten central states of the US, which included Kansas. Table 1 reports movement parameters among beef farms (*B*) and preslaughter farms (*P*) of sizes 1–19, 20–199, and 200-up number of cattle. Distances are grouped in ranges such that the index in variable *d* is the maximum distance in miles of the range with the minimum defined by the previous level; for example, *d*_500_ indicates a distance between the two county centers falling between 200 and 500 miles. Since the closest range, *d*_0_, is assigned to any pair of counties with centers less than 10 miles apart, as well as each county with itself, we have used these probabilities for our scenario in Riley County, KS. Parameters in Table 1 represent scaled probabilities of movement within a week’s duration. The scaling factor is 10^3^.

**Table 1:** Estimated Cattle Movement parameters *p* x 10^3^, beef to beef and beef to preslaughter.

Farm size and type		Probability p x 10^3^				
Origin farm	Destination farm	*d*_0_	*d*_100_	*d*_200_	*d*_500_	*d*_1000_
*B*(1–19)	*B*(1–19)	0	0	0	0	0
*B*(20–199)	*B*(1–19)	0	0	0	0	0
*B*(200-up)	*B*(1–19)	0.00424301	0	0	0	0
*B*(1–19)	*B*(20–199)	0	0	0	0	0
*B*(20–199)	*B*(20–199)	0	0	0	0	0
*B*(200-up)	*B*(20–199)	1.01066499	0	0	0	0
*B*(1–19)	*B*(200-up)	0	0	0	0	0
*B*(20–199)	*B*(200-up)	0	0	0	0	0
*B*(200-up)	*B*(200-up)	1.297178111	0	0	0	0
*B*(1–19)	*P*(1–19)	0.100731885	0.001370626	0	0	0.0042165
*B*(20–199)	*P*(1–19)	0	0	0	0	0
*B*(200-up)	*P*(1–19)	0	0.000119848	0	0.000241394	0
*B*(1–19)	*P*(20–199)	0.255802297	0.006266095	0.000343754	0	0.0004223
*B*(20–199)	*P*(20–199)	0	0	0	0	0
*B*(200-up)	*P*(20–199)	0.144546061	0	0.000663091	0.000306429	0
*B*(1–19)	*P*(200-up)	0	0	0	0	0
*B*(20–199)	*P*(200-up)	0	0	0	0	0
*B*(200-up)	*P*(200-up)	0.633105311	0	0	0.000817912	0

Using probabilities of Table 1, we derive the contact network shown in Fig 4.This contact network is not based on any model, but rather is based on the above-estimated probabilities of movements for the distance range labeled *d*_*0*_.

#### Mosquito abundance and vectorial capacity estimate

We present in Table 2 vectorial capacity estimates calculated from 22 annual (1993–2014) real-world collections of select potentially high-risk US mosquitoes from the Fort Riley area competent for transmission of RVFV based on laboratory studies. Mosquito collection data were available from Fort Riley dating back to 1975; however, the 22 year 1993–2014 period was the largest unbroken collection record in the full 39 year data set, also with the most consistent group of months sampled each year. Although collections had been conducted with a variety of mosquito traps across the 39 year record, the New Jersey light trap was by far the most consistently used sampling equipment and we restricted all analyses to data gathered with this trap in the 22 year subset.

**Table 2:** Estimated mosquito vectorial capacity.

*Aedes vexans*	*Culex pipiens*	*Culex salinarius*	*Culex tarsalis*	*Psorophora columbiae*
0.0134	0.0055	0.0107	0.0200	0.0022

The Garett-Jones [35] equation for estimating vectorial capacity *C* is given as follows:

$$C=\frac{ma^{2}p^{n}b}{-\ln(p)}$$

where *m* = mosquito vector density, with respect to the host, *a* = daily probability of host being fed upon, *p* = probability of daily survival, *n* = length of extrinsic incubation period in days, and *b* = vector competence (proportion of mosquitoes able to transmit RVFV [26, 28]). Data were not available for *a* nor *n*, and although data were available for mosquito density from New Jersey light trap collections, we did not have data to calculate this density with respect to host density, *m*. Also, we did not have directly-measured data on the probability of daily survival of the focal mosquito species, *p*. However, borrowing some of the structure of the Garrett-Jones equation we proceeded to estimate vectorial capacity using vector competence data from Golnar et al. [26] and Turell et al. [28] laboratory studies combined with available parameters that could be derived from real world Fort Riley mosquito surveillance, namely, relative mosquito vector density and an index of sustained population survival.

Prior to analysis, all mosquito surveillance data were log-transformed (ln) [36, 37]. Once log-transformed, all 1993–2014 Fort Riley mosquito surveillance data were reduced to a monthly ‘trap night index’; i.e., the number of females of a given species collected per night per trap across all traps in a given month. The following five focal species, *Ae*. *vexans*, *Cx*. *pipiens*, *Cx*. *tarsalis*, *Cx*. *salinarius*, and *Ps*. *columbiae*, were selected from the long-term Fort Riley mosquito surveillance record using the criteria that (a) they had been identified in laboratory studies [26] as potential US mosquito vectors of RVFV, (b) they are expected to feed on cattle, and (c) >1000 female specimens had been collected across the full 1975–2014 sampling record at Fort Riley. We then calculated the trap night index anomaly value for each species in each month; i.e., the numerical difference between the trap night index for a given month for a given species and that species’ long-term mean trap night index for that month across the 22-year record. Next, we plotted the accumulated anomaly for each species in each sample year across the 22-year record. For example, plot the anomaly value for April in April, the anomaly values for April + May in May, the anomaly values for April + May + June in June, and so on. This derived index provides information on the extent to which a population is not only larger than normal but also whether the increase is sustained across the sampling (and disease transmission) season. The trap night index anomaly data and the accumulated anomaly data were used to estimate vectorial capacity as follows:

*Vector density m*. We assume that host density remains constant, and only estimate the extent that populations emerge at above-normal levels across multiple samples. For instance, if a species exists at above-normal densities across the majority of sample years, then any year that RVFV may arrive at a location in the study area we can assume that this species is present at an above-normal density. This higher *m* would contribute to a higher vectorial capacity compared to another species that may tend to exist at more normal or below-normal densities across the sample years (lower *m*). We calculated *m* as (mean positive anomaly value) * (frequency of months with positive anomaly values), simplified to (sum of positive anomalies) / (number of months sampled).

*Probability of seasonal survival p*. This is estimated by the extent that populations show a tendency to emerge at incrementally higher above-normal levels across multiple samples. We substitute probability of daily survival with this index of the presence of the species throughout the season based on long term data. For example, if a species tends to have an increasing trend of above-normal population density each season across the sampled years, then any year that RVFV may arrive at a location in the study area, we can assume that abundant individuals of this species would live long enough to feed on an infected cow, permit viral replication and dissemination, and be able to transmit the virus to a naïve cow. This higher *p* would contribute to a higher vectorial capacity compared to another species that may tend to be constant or decline in numbers through a season in a given year, and so have a lower relative likelihood of individual mosquitoes surviving through the transmission cycle. We calculated *p* as (mean of slopes of positive accumulated anomalies) * (frequency of years with positive accumulated anomalies), simplified to (sum of slopes of positive accumulated anomalies) / (number of years sampled).

*Vector competence b*, is the proportion of mosquitoes able to transmit RVFV; obtained from Golnar et al. [26] and Turell et al. [28]

With these parameters we estimated vectorial capacity, *C*, using the equation *C* = *m* * *p* *b / ln (*p*).

Also in Table 3, we report data on the community structure of these species, given as the long term mean relative abundance of each focal mosquito species, so that we can compute a composite effective vectorial capacity index across several species simultaneously.

**Table 3:** Mosquito community structure.

*Aedes vexans*	*Culex pipiens*	*Culex salinarius*	*Culex tarsalis*	*Psorophora columbiae*
0.3775	0.0605	0.1733	0.2022	0.1865

The five focal species were sampled concurrently across the 22-year 1993–2014 record, which we used to calculate the relative abundance of the five species from the total of the number of females sampled across all species. For an ecological region containing two or more species simultaneously, an effective vectorial capacity would be the average of the respective vectorial capacities weighted by their community structure relative abundance.

## Simulation Results and Discussion

For these simulations, we use GEMF tool to understand the relationship between values of *β* (representing vectorial capacity) and *k* or *D*_*min*_ (representing contacts through movements) and the average size of a hypothetical outbreak of RVF in a simulated environment around Riley County KS.

To test the effect of different contact patterns, we use two models: exponential and power law. In the exponential model, links between distant nodes have low probability whereas, in the power law model, network links at high distances are possible.

According to the exponential model, we generated a network where the cattle within a farm are all connected, and the links between different farms were created with probability *p* = *e*^-*kd*^, where *d* is the distance between two farms and *k* is a parameter. The network was then subject to extensive simulation for given intra-farm distances (*d*) and a set of parameter *k* to find the average number of infected animals. The standard deviation and the confidence interval was also calculated for each parameter set (see Supporting Information).

**Fig 7:**
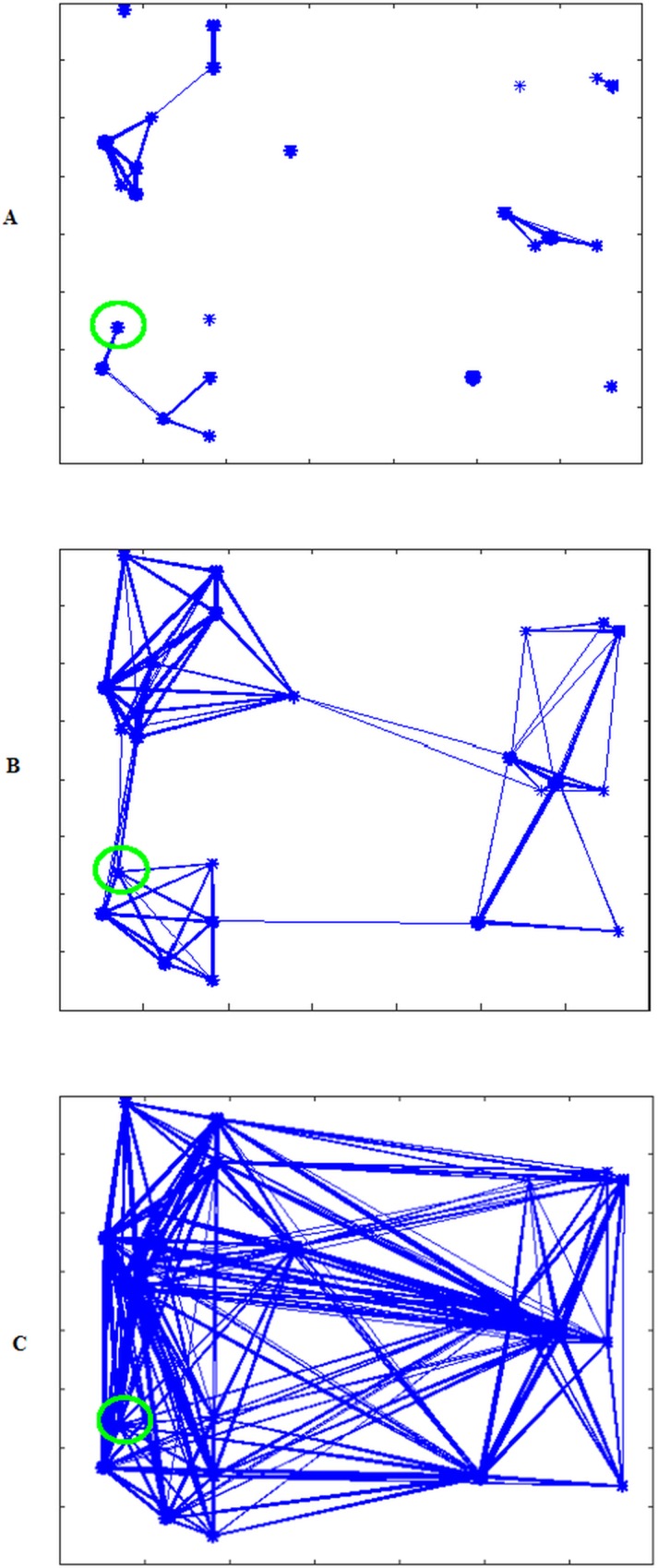
Contact network for a cattle population in 27 farms and inter-farms links generated with an exponential model. (A) Contact network for *k* = 1 km^-1^ (*G*_*exp*100_). (B) Contact network for *k* = 0.4 km^-1^ (*G*_*exp*40_). (C) Contact network for *k* = 0.2 km^-1^ (*G*_*exp*20_).

For *k* = 1 km^-1^, the chosen network *G*_*exp*100_ is shown in Fig 7A. It has *L* = 2,225,503 links, out of a maximum of *L* = 29,541,141 links. In this case, the infection does not spread until *β* ≥ 0.6 x 10^−3^. Then, initially the infection spreads only within the farm of the first infected cow (i.e., farm 1; green circles in Fig 7A–7C), and later it spreads to other farms nearby. Because there are few links the infection remains confined to a few farms and the maximum average final infection size is 17%.

For *k* = 0. 4 km^-1^, we generate a network *G*_*exp*40_ that has, as expected, an increased number of links (L = 2,409,077). Fig 7B shows that in this network all farms belong to a single connected network component, but it also shows that there are three groups of well-connected farms only connected by a few links. For this network, when *β* is large enough, the entire population can be infected, i.e. the maximum average final infection size is 100%. Finally, setting the parameter *k* = 0.2 km^-1^, we create an even more dense network *G*_*exp*20_ with L = 2,966,050, depicted in Fig 7C. Comparing the outbreak sizes in *G*_*exp*40_ and *G*_*exp*20_, we observed that even a lower vectorial capacity is enough for the infection to invade the whole network in the case of high movement levels of *G*_*exp*20_.

In Table 4 we summarize the final average number of infected cattle for a variable *β* parameter, for these three networks.

**Table 4:** Final average infection size (percentage of cattle population).

*β* x 10^−3^	*G*_exp100_	*G*_exp40_	*G*_exp20_
0	0	0	0
0.1	0	0	0
0.2	0	0	0
0.3	0	0	0
0.4	0	0	4
0.5	0	0	6
0.6	0	0	6
0.7	1	3	6
0.8	1	4	9
0.9	1	4	17
1	1	5	16
2	1	6	70
4	2	16	80
6	4	28	90
8	5	40	90
10	6	42	90
12	8	55	100
14	8	53	100
16	8	56	100
18	9	53	100
20	9	70	100
40	11	76	100
60	14	83	100
80	11	92	100
100	11	92	100
120	11	89	100
140	11	89	100
160	11	93	100
180	11	96	100
200	16	96	100
220	16	100	100
240	16	100	100
260	17	100	100
280	17	100	100
300	17	100	100

**Fig 8:**
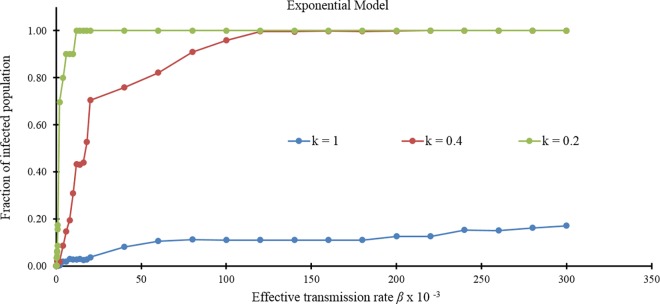
The average fraction of infected population for increasing parameter *β* for *G*_exp100_, *G*_exp40,_ and *G*_exp20._

We assume that the vectorial capacity for *Cx*. *tarsalis* from Table 2 (0.0200) corresponds to the row outlined in gray in Table 4. The measured vectorial capacity could produce very large outbreaks when the level of movements is high as in *G*_*exp*20_. Table 4 shows how the increase in the fraction of the infected population as a function of increased vectorial capacity shows nonlinearity and the presence of plateaus, which is graphically summarized in Fig 8 for increasing values of *β*.

**Fig 9:**
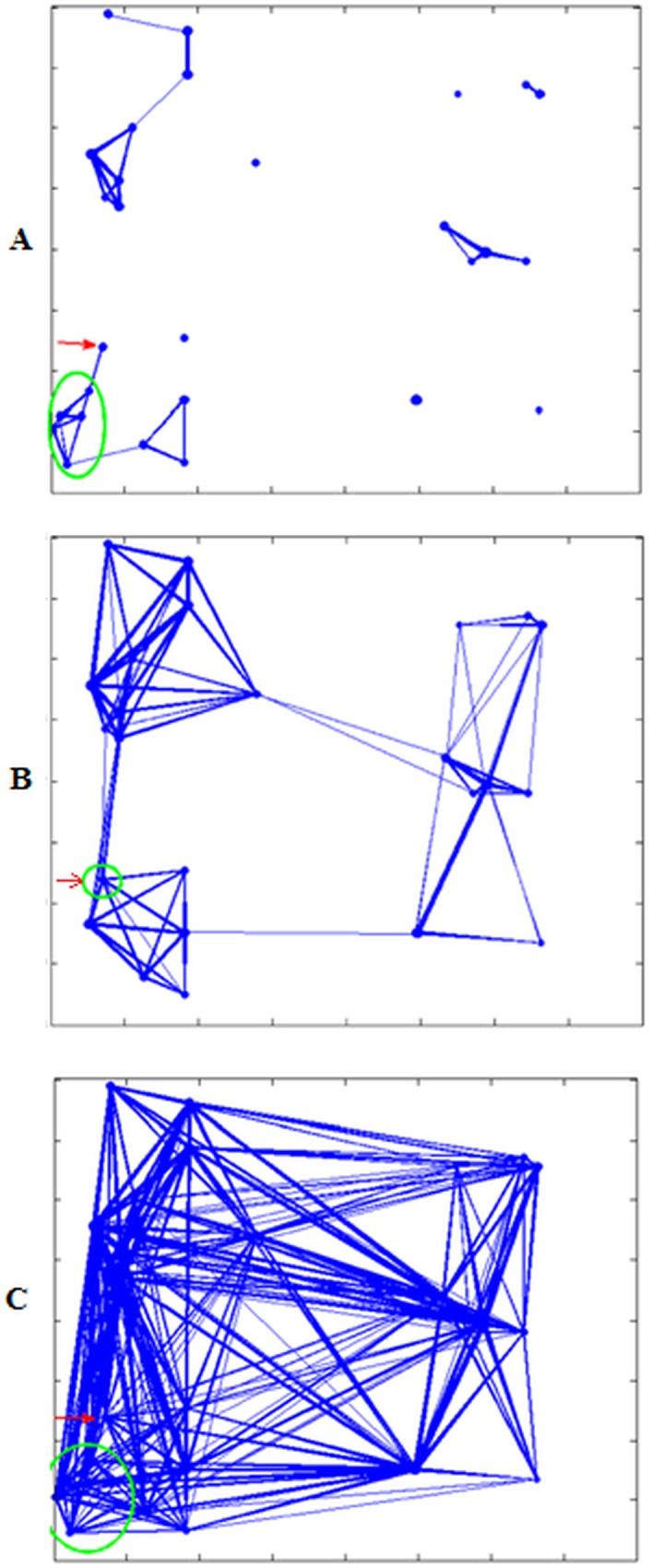
Contact network for a cattle population in 27 farms. (A) Contact network for *k* = 1 km^-1^ (*G*_*exp*100_). (B) Contact network for *k* = 0.4 km^-1^ (*G*_*exp*40_). (C) Contact network for *k* = 0.2 km^-1^ (*G*_*exp*20_). One large farm is divided in 5 communities and inter-farms and inter-community links are generated with exponential probability.

In the next set of simulations, we test the effect of changing the structure of the largest farms into smaller separated communities. Through the use of our individual-based model, we can divide large farms into subgroups. In particular, farm 1 (i.e., the farm with the initial RVF infection) has 500 cattle that, in previous simulations, were in full contact. Now we divide this single farm into five communities (red circles in Fig 9A–9C) each with 100 cattle, reducing intra-farm contact. Very few links are left among the communities, to account for the rare transfer of cattle from one community to another. The initially infected animal is located on the same farm (green circles in Fig 9A–9C) as in previous simulations. Similarly, as in the previous scenarios, we generate three networks with the exponential model, *G*_*exp*100_, *G*_*exp*40_, and *G*_*exp*20_. Fig 9A–9C show the topologies of these three networks.

**Fig 10:**
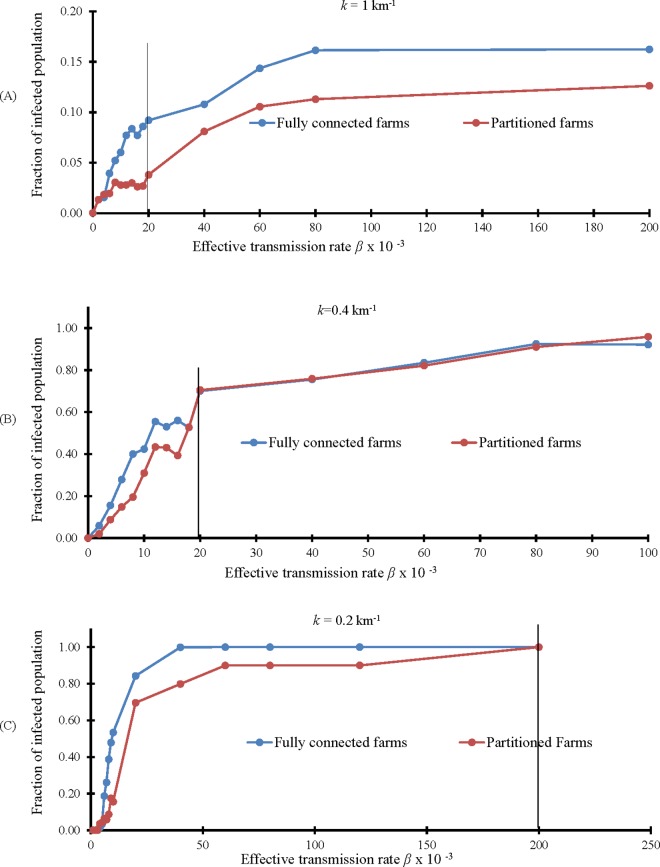
Comparison between fractions of infected with each farm fully connected or partitioned. (A) Comparison between fractions of infected for *G*_*exp*100_. (B) Comparison between fractions of infected for *G*_*exp*40_. (C) Comparison between fractions of infected for *G*_*exp*20_. For all figures, the vertical black line indicates vectorial capacity 0.02.

With these new three networks, we run our model to compute the average final size of the epidemic, and compare the two scenarios with and without the large farm separation, called Partitioned farm. Results are summarized in Fig 10A–10C.

We can observe that for the measured vectorial capacity, *β* = 0.02, partitioning the farm into several parts has the effect of reducing the total size of the outbreak when the level of movement is low and when the vectorial capacity is relatively small. In general, the effect of the farm partitioning brings a reduction of the final infection size for specific ranges of *β*.

**Fig 11:**
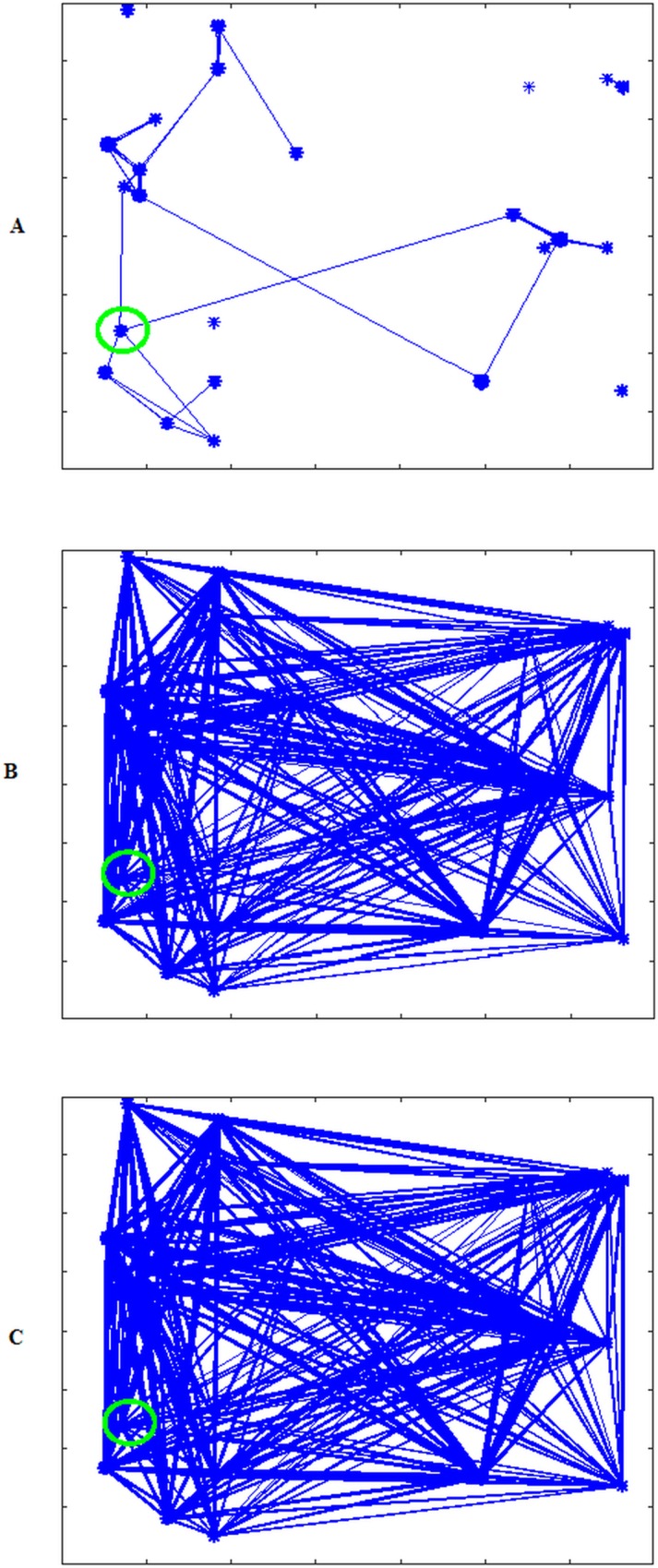
Contact networks generated using a power law probability distribution. (A) Contact network for *G*_*pl*100_ with number of links similar to *G*_*exp*100._ (B) Contact network for *G*_*pl*40_ with number of links similar to *G*_*exp*40._ (C) Contact network for *G*_*pl*20_ with number of links similar to *G*_*exp*20._

Next, we generate contact networks based on a power law. We named these networks *G*_*pl*100_, *G*_*pl*40_, and *G*_*pl*20_ corresponding to exponential networks *G*_*exp*100_, *G*_*exp*40_, and *G*_*exp*20_. The difference between the exponential and power law models is pronounced in Fig 11A, as this network has more links between distant farms (compare with Fig 9A). In the latter case of Fig 11C, the difference is less visible because many links already exist between distant farms.

**Fig 12:**
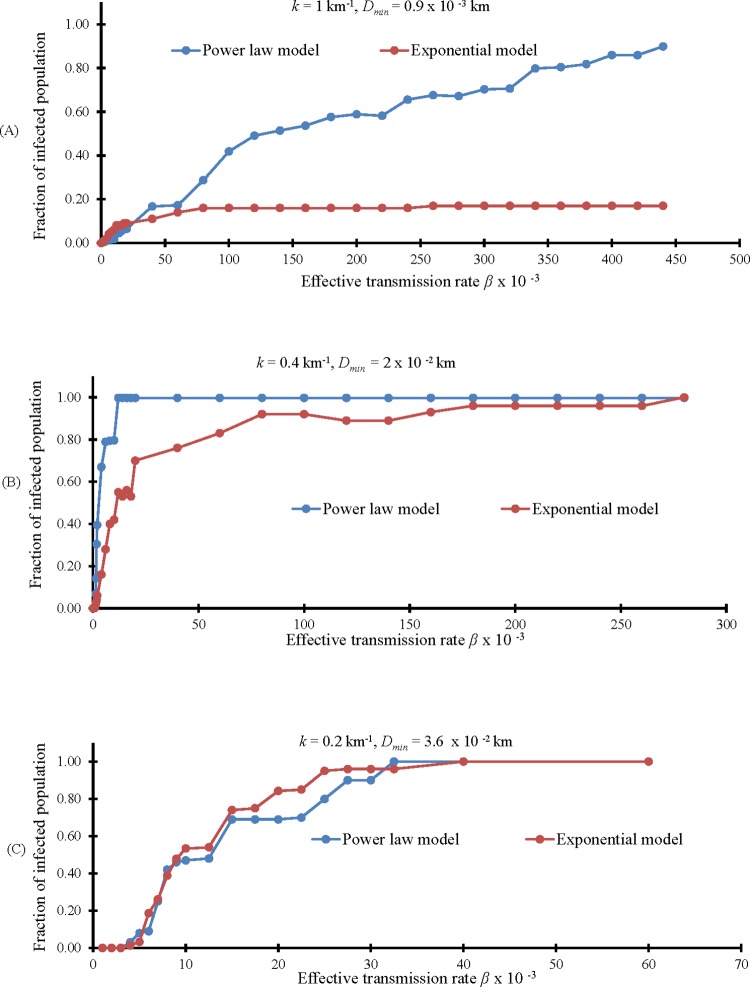
Comparison between fraction of infected population for Exponential and Power Law model. (A) Comparison between fractions of infected in *G*_*pl*100_ and *G*_*exp*100._ (B) Comparison between fractions of infected in *G*_*pl*40_ and *G*_*exp*40._ (C) Comparison between fractions of infected in *G*_*pl*20_ and *G*_*exp*20._

In Fig 12, a comparison between average fractions of infected population obtained by using exponential and power law models is presented. Using a power law model, links that connect distant farms are generated even when the network has a small number of links such as in *G*_*exp*100_ (Fig 11A). As a consequence, the infection does not remain confined in few adjacent farms as in the exponential case, but it spreads to farms in other areas as shown in Fig 12A. In the second case (Fig 11B), the power law network has more links between distant farms than the exponential case, so the infection expands to a larger area, as seen in Fig 12B. In the last case, the situation is almost similar because also the exponential network already has many links between distant farms (Fig 12C).

Finally, we consider the contact network *G*_*data*_ from the estimated movement rates in [24], shown in Fig 4. In this network we consider two cattle classes: beef and preslaughter. No dairy farms were included in our scenario. Performing and averaging several simulations for this contact network and for a value of *β* = 0.02—the reported vectorial capacity for *Cx tarsalis* in Riley County, Kansas, from Table 2—the corresponding average infection size is around 91%. This same contact network was used for other vectorial capacities calculated from all five mosquito species in Table 2 and mosquito community structure from Table 3. To find the composite effective vectorial capacity we summed each mosquito species’ vectorial capacity weighted by their fraction of presence in the environment. This value of vectorial capacity is *β* = 0.01287 and in this case the average infection size is 82%. The results of these simulations suggest that the estimated movement parameters are consistent with movement network models of high connectivity, which can potentially produce a large-size epidemic.

## Conclusions

In this paper, we propose a parsimonious individual-based network model to evaluate the impact of several interventions on RVF outbreaks in a cattle population in Kansas, US. The single parameter *β* represents the vectorial capacity of a mosquito species. Since we only focused on Riley County in Kansas and for a limited period, we kept *β* constant in our simulations. However, this parameter can be easily set to vary in time, to represent annual variability and seasonality, and it can vary in space, to represent different eco-regions. Through our simulations, we can see that when we decrease vectorial capacity, the average final size of the outbreak also decreases. However, this relationship is non-linear, exhibiting plateaus and abrupt transitions. The second aspect that we study in this paper is how beneficial it might be to divide a large farm into several smaller farms. Our simulations show that this strategy can be very beneficial when the vectorial capacity and the level of movements are relatively small. We would like to point out that dividing the farm implies reduced opportunity for mosquitoes to contact cows across patches that are separated by distance, or by mosquito control interventions such as pesticide-treated barriers [38, 39]. Comparing exponentially and power-law generated contact networks with the minimum number of links, the epidemic size reaches 90% of the population with the power law model, while for the exponential model it remains constant at 17%. Increasing the number of links, we can observe more links among distantly separated farms in the power law network. In this way, the infection spreads more easily than in the exponential case. Finally, when the number of distant links is high, both power law and exponential models exhibit similar infection propagation behavior.

One factor that we did not include in this model is the potential for RVFV spreading through wild ungulate populations, such as white-tailed deer (*Odocoileus virginianus*). Certainly, deer may be observed co-grazing with cattle in many areas of the US and their potentially unlimited contact with mosquitoes, unrestricted movements, and, in places, vast population sizes definitely mark them as a potentially significant factor in RVFV epidemiology in the US. However, the relative susceptibility of US wild ungulates to RVF infection and their levels of viremia are poorly known or unknown, compared to the acute susceptibility to RVF infection and high viremias that have been observed among European breeds of domestic livestock in RVF-endemic regions over decades [40–43]. We purposely chose to restrict the model to industrial cattle populations because the well-defined network structure and the potential information at the individual level for cattle movements make the system very appropriate to be studied with our approach. Future work will consider the interaction of the domestic cattle network with the wild life through an interconnected network approach.

In conclusion, looking at the simulations in more detail, we observe that an RVFV outbreak could be contained in three ways. The first is through reduction of the population of RVFV vector mosquitoes with larval or adult mosquito control methods. The second is by restricting animal movements. In other words, with less movement, the infection may remain confined, for example, in a small number of farms without contaminating a larger area. The third solution is to maintain a lower infection size, as seen by comparing the blue curve and the red curve in Fig 9, accomplished by dividing the cattle into groups within a farm. If a cow is infected within a subdivided group, it can infect mosquitoes that in turn can infect other cattle within that group, but not necessarily cattle in other groups or, later, other farms.

## Supporting Information

S1 Fig The fraction of the infected population for increasing *β* with 95% confidence interval. (A) The fraction of the infected population for exponential network G_*exp100*_. (B) The fraction of the infected population for exponential network G_*exp40*_. (C) The fraction of the infected population for exponential network G_*exp20*_. With increasing*β*, the fraction of infected is showing an increasing trend until reaching a maximum point (close to 1) for all stochastic simulation. This is because *β* is the vectorial capacity of mosquitoes and it controls the chain of pathogen communication from cattle to cattle in the whole contact network.
(TIF)

S2 Fig The sensitivity of parameter *β* with increasing fraction of the infected population. (A) Sensitivity for exponential network G_*exp100*_. (B) Sensitivity for exponential network G_*exp40*_. (C) Sensitivity for exponential network G_*exp20*_. The sensitivity analysis is performed computing the derivative of the fraction of the infected population as a function of *β* (α- y axis) corresponding to the given fraction of the infected population (x axis) (S2A–S2CFig). As the fraction of infected is increased, the sensitivity of *β* is decreasing, because a large number of susceptible population is required for maintaining a high sensitivity. However, with an increasing fraction of the population infected, the fraction of the susceptible population decreases reducing the sensitivity of *β*.
(TIF)
