# Supplementary figures and images for: Biologically Informed Individual-Based Network Model for Rift Valley Fever in the US and Evaluation of Mitigation Strategies
Source: PLoS One. 2016 Sep 23;11(9):e0162759. doi: 10.1371/journal.pone.0162759 (PMC5035079; doi:10.1371/journal.pone.0162759)

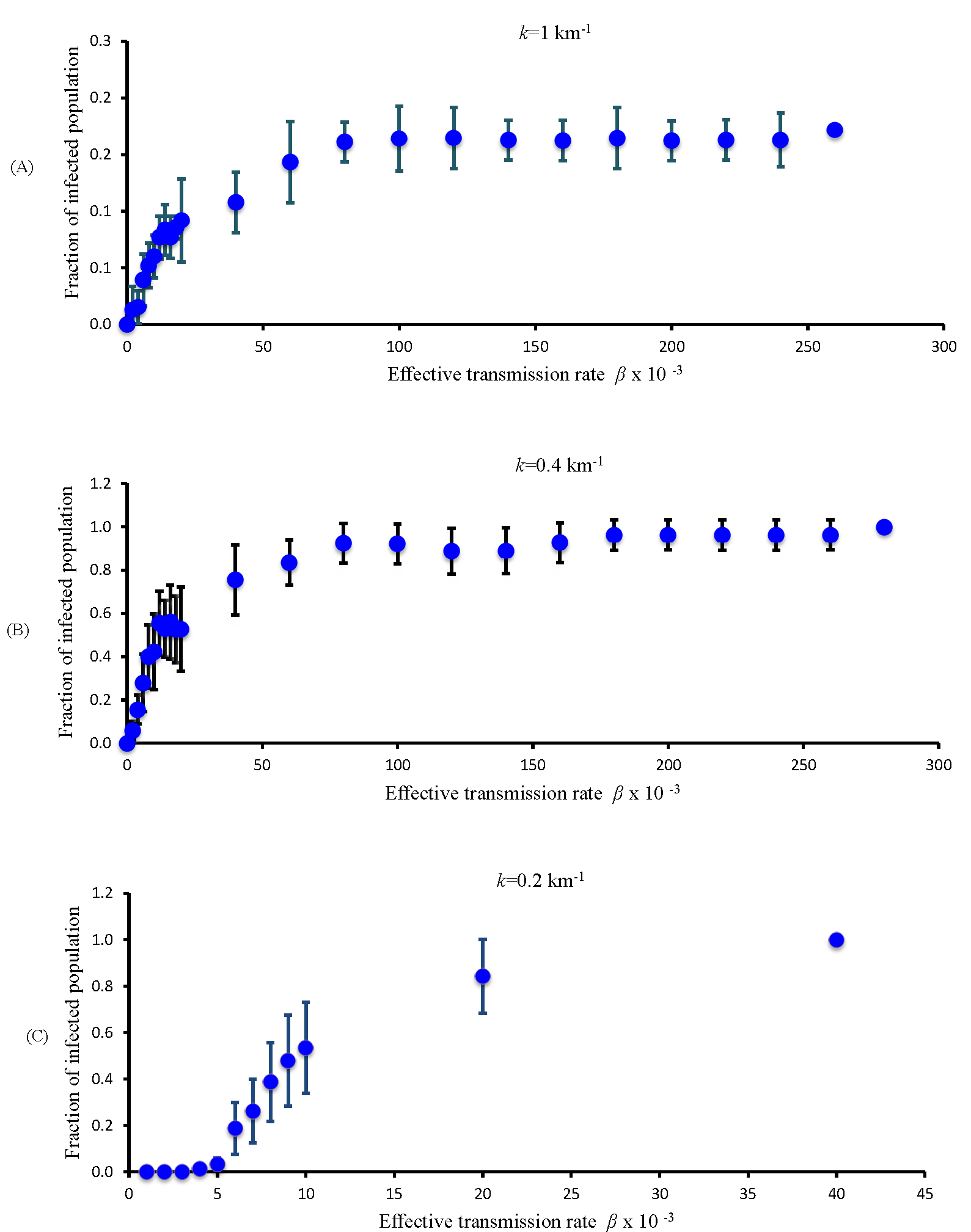

Supplement: S1 Fig — (A) The fraction of the infected population for exponential network Gexp100. (B) The fraction of the infected population for exponential network Gexp40. (C) The fraction of the infected population for exponential network Gexp20. With increasingβ, the fraction of infected is showing an increasing trend until reaching a maximum point (close to 1) for all stochastic simulation. This is because β is the vectorial capacity of mosquitoes and it controls the chain of pathogen communication from cattle to cattle in the whole contact network. (TIF) [file pone.0162759.s001.tif]

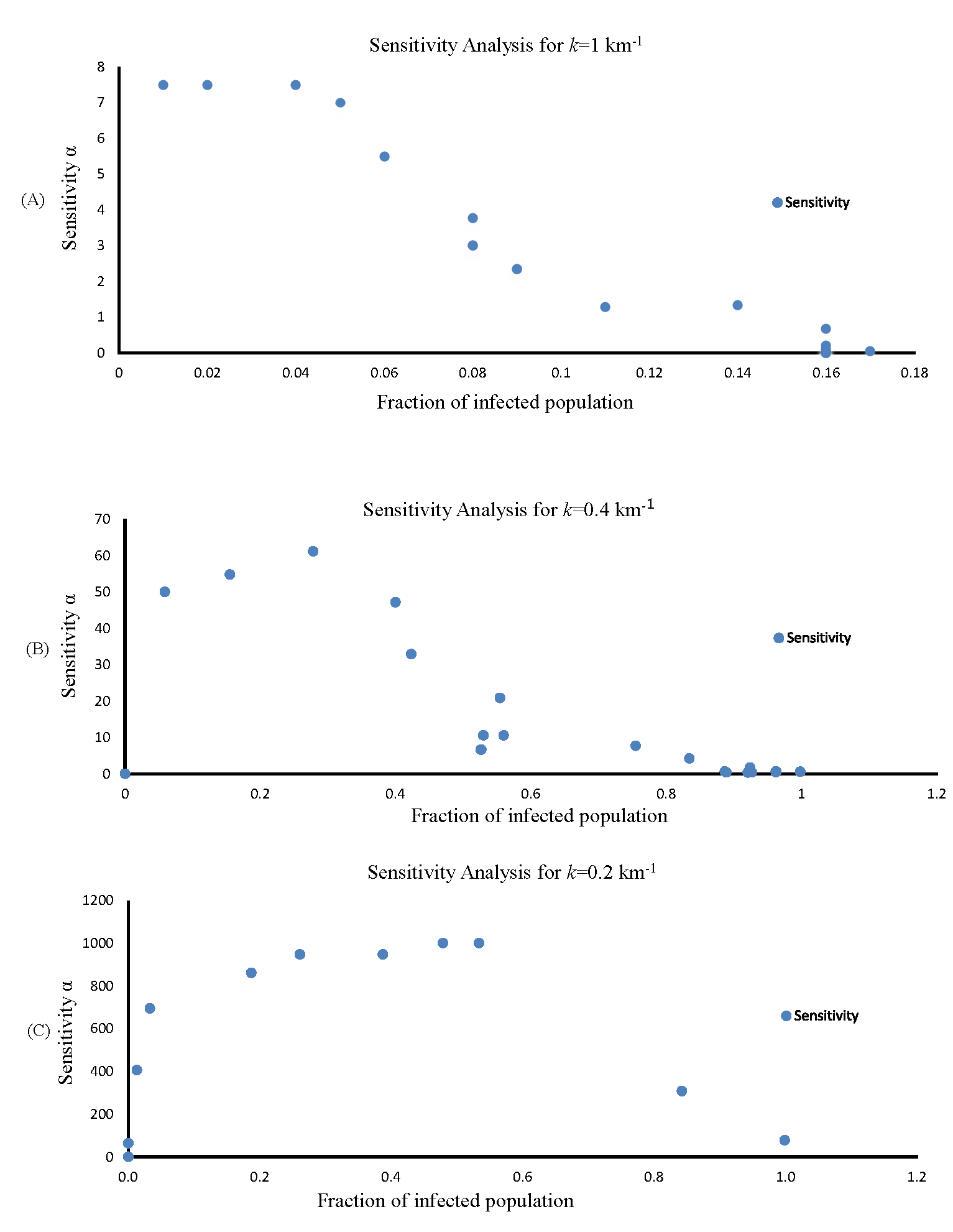

Supplement: S2 Fig — (A) Sensitivity for exponential network Gexp100. (B) Sensitivity for exponential network Gexp40. (C) Sensitivity for exponential network Gexp20. The sensitivity analysis is performed computing the derivative of the fraction of the infected population as a function of β (α- y axis) corresponding to the given fraction of the infected population (x axis) (S2A–S2CFig). As the fraction of infected is increased, the sensitivity of β is decreasing, because a large number of susceptible population is required for maintaining a high sensitivity. However, with an increasing fraction of the population infected, the fraction of the susceptible population decreases reducing the sensitivity of β. (TIF) [file pone.0162759.s002.tif]
